# Is Resistance to Dolutegravir Possible When This Drug Is Used in First-Line Therapy?

**DOI:** 10.3390/v6093377

**Published:** 2014-08-27

**Authors:** Thibault Mesplède, Mark A. Wainberg

**Affiliations:** McGill University AIDS Centre, Lady Davis Institute for Medical Research, Jewish General Hospital, 3755, Ch. Côte-Ste-Catherine, Montréal, QC, H3T1E2, Canada; E-Mail: tibo_mes@hotmail.com

**Keywords:** HIV integrase, dolutegravir, resistance, R263K, viral fitness, eradication

## Abstract

Dolutegravir (DTG) is an HIV integrase inhibitor that was recently approved for therapy by the Food and Drug Administration in the United States. When used as part of first-line therapy, DTG is the only HIV drug that has not selected for resistance mutations in the clinic. We believe that this is due to the long binding time of DTG to the integrase enzyme as well as greatly diminished replication capacity on the part of viruses that might become resistant to DTG. We further speculate that DTG might be able to be used in strategies aimed at HIV eradication.

## 1. Introduction

Current HIV therapy usually involves the use of three antiretroviral (ARV) drugs in combination, often as part of a simplified regimen. Indeed the introduction of triple ARV therapy in 1996 has since led to rates of therapeutic success that have increased to over 90%, based on suppression of plasma viremia to below 50 copies of viral RNA/mL. This progress is attributable to several facts: 1. Dosing regimens have become simplified, often because of the use of co-formulations, some of which only need to be taken once-daily; this has greatly enhanced rates of adherence to ARV regimens; 2. Pill regimens have become far less toxic and more tolerable over time; this has also promoted adherence as well as diminished the likelihood of development of HIV drug resistance against individual drugs [1,2]; 3. The drugs used in therapy are now more potent than the compounds that were in use only 10 years ago.

To be sure, the use of ARVs in first line regimens has always been associated with some degree of drug resistance and treatment failure. Over the past several decades, scientists have catalogued a wide array of drug resistance mutations that are located within each of the reverse transcriptase, integrase and protease enzymes of HIV-1 that are the targets of HIV therapy, and have documented how each of these mutations may lead to diminished likelihood of a favorable clinical response to each ARV, both in therapy and in cell culture [1]. In addition, phase III clinical trials that led to the approval of each of the ARVs now used for therapy also provided valuable information on the types of mutations that were most likely to be identified in the virus in the aftermath of viral rebound. This includes members of the integrase strand transfer inhibitor (INSTI) family of drugs such as raltegravir (RAL) and elvitegravir (EVG) [3,4,5,6,7]. 

Recently, a drug termed dolutegravir (DTG) has been studied in phase III investigations and has yielded the most robust results ever obtained in HIV clinical trials [8]. First, approximately 88% of patients who received DTG in these studies attained suppression of viral load to <50 copies RNA/mL and, in addition, none of the individuals in these studies possessed a single drug resistance-related mutation that was associated with either DTG or the nucleoside drugs that were used together with DTG as a part of therapy. It should be noted, however, that approximately 10%–15% of patients in the trials did not respond to therapy and possessed detectable levels of viral load in plasma, perhaps for reasons of non-adherence [9,10].

Of course, resistance against boosted protease inhibitors (PIs) after virological failure (VF) was also very rare but this has been primarily investigated for mutations in the viral protease (PR) gene (1). It is possible, of course, that mutations at gag cleavage sites may have been present in certain of these cases. In addition, the M184V mutation, associated with resistance to 3TC, was present in some cases of failure involving boosted PIs.

## 2. Viral Fitness Prevents HIV-1 from Evading Dolutegravir Pressure

**Figure 1:**
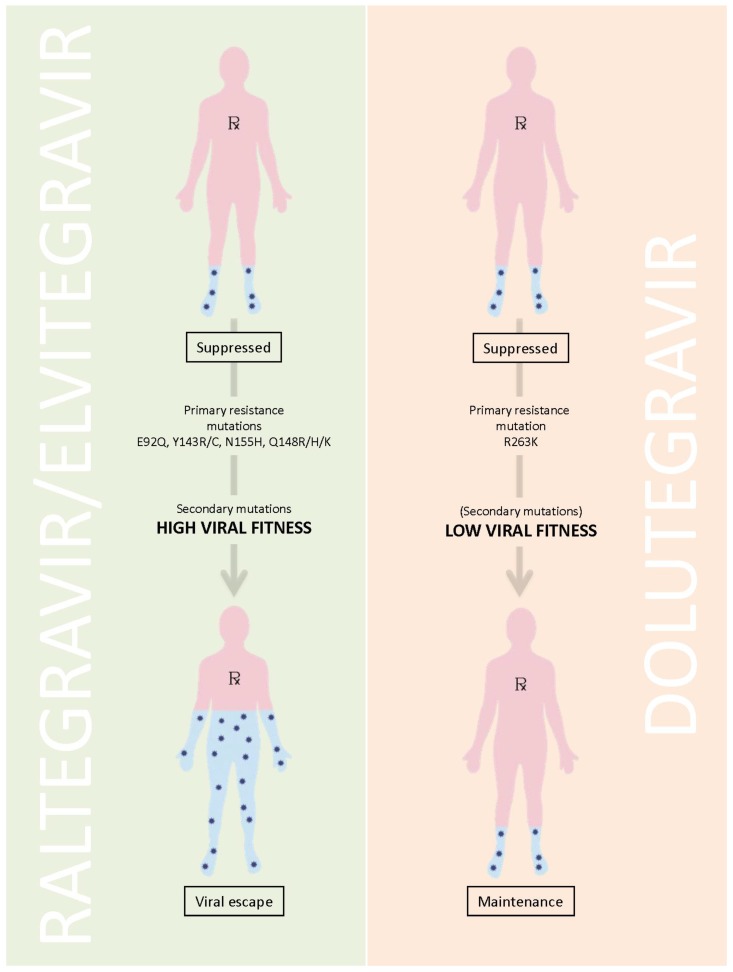
Schema of Low Viral Fitness due to the R263K Resistance Pathway.

The question is how to explain these results. Among the hypotheses that have been advanced is that viruses that become resistant to DTG may be relatively replication incapacitated and cannot efficiently grow; hence, such variants might not be detectable in patient plasma [11] (Figure 1). It is known, for example, that DTG can select a mutation at position R263K in the integrase gene in tissue culture and that this mutation diminishes both viral replication capacity as well as the enzymatic activity of the integrase enzyme [12]. Although this is not unusual, it should be noted that similar results were also obtained with the two other approved integrase inhibitors EVG and RAL [11]. Indeed, in the case of the latter two compounds, the presence of an initial substitution was often quickly followed by the appearance of a second mutation that had the dual effect of increasing the level of drug resistance, often to a level that might preclude any further clinical benefit from the drug, while simultaneously restoring viral replication capacity to close to that of wild-type viruses (Table 1). However, the secondary mutations that were selected by DTG only modestly increased overall levels of resistance against the drug but simultaneously impacted even more adversely on the ability of the virus to grow, often resulting in impairment of >80%, and this was accompanied by a further diminution in the activity of HIV integrase in biochemical assays [11,12]. These findings may be due in large part to the fact that the ability of DTG to bind to the integrase enzyme is extremely long and exceeds by at least several fold the ability of either RAL or EVG to achieve similar binding [13].

**Table 1:** Resistance pathways for each of dolutegravir (DTG), raltegravir (RAL), and elvitegravir (EVG).

EVG/RAL	Dolutegravir
**E92Q pathway**	
E92Q	
T66I/E92Q	
E92Q/S153A	
E92Q/H51Y/L768V	
**N155H pathway**	
N155H	
L74M/N155H	
E92Q/N155H	
**Y143 pathway**	**R263K pathway**
Y143C	R263K
Y143R	M50I/R263K
T97A/Y143C	H51Y/R263K
T97A/Y143R	E138K/R263K
L74M/T97A/Y143G	
L74M/T97A/E138A/Y143C	
**Q148 pathway**	
Q148H	
Q148K	
Q148R	
E138K/Q148H	
E138K/Q148K	
E138K/Q148R	
G140S/Q148H	
G140S/Q148K	
G140S/Q148R E138A/G140S/Y143H/Q148H	

It should be stated that secondary and/or tertiary drug resistance mutations often play a compensatory role in regard to replication for many microorganisms besides HIV, including bacteria that are resistant to numerous antibiotics as well as viruses that display resistance against specific antiviral drugs. Compensatory mutations in HIV that simultaneously augment viral replication while increasing overall levels of drug resistance have been documented for members of each of protease inhibitors (PIs) as well as the nucleoside reverse transcriptase inhibitor (NRTI) and non-nucleoside RT inhibitor (NNRTI) families of drugs [1]. However, no such mutation has been identified for DTG, representing a unique observation that is bolstered by the results of tissue culture selection experiments that have yielded only two distinct mutations that diminish viral replicative capacity but never a third compensatory mutation [11] over more than four years of selection pressure in culture.

## 3. Can Dolutegravir Be Used in Strategies Aimed at HIV Eradication? 

Accordingly, we should wonder what will happen if viruses that are resistant to DTG cannot be compensated by additional mutations within integrase and if such viruses are truly at a severe replication disadvantage in comparison with wild-type HIV. Would such a result take on even greater significance if it turned out that DTG can retain clinically significant antiviral activity, despite the presence of one or two drug resistance mutations? Such a scenario is indeed suggested by the fact that the level of resistance conferred against DTG by the combination of two such mutations within integrase is <10-fold and that biochemical results have shown that the ability of DTG to bind to the integrase enzyme and remain associated with it is very long, *i.e.*, >60 hours. Moreover, the R263K mutation only diminished this level of binding by about 50% [13,14] which is still far longer than the binding affinity half-life of RAL and EVG for wild-type integrase. This raises the possibility that the development of low-level resistance against DTG in first-line therapy might not have adverse virologic or clinical consequences. However, it should also be noted that DTG was only approved for treatment in the USA approximately one year ago and that all of the clinical data that pertain to this compound have been obtained as part of clinical trials. Support for this concept will only accrue after DTG is widely prescribed outside of clinical trial settings, including under conditions in which a far greater degree of non-adherence to treatment can be expected. At the present time, the data suggest that patients who may become resistant to DTG will still respond to RAL, but further clinical experience will be needed to substantiate this point.

How could this hypothesis be tested? First, a study could be contemplated in which DTG is employed as monotherapy in treatment-naive subjects, even though we would prefer that proof-of-concept results first be obtained in relevant animal models. If the results obtained are similar to those observed in the phase III clinical trials, a partial validation of the hypothesis to explain the absence of resistance in the phase III trials will have been obtained. It goes without saying that such a monotherapy study would need to be accompanied by intense virologic monitoring for resistance mutations, that should include the use of ultrasensitive sequencing for identification of DTG resistance mutations in the DNA of patient peripheral blood mononuclear cells as well as in the RNA of patient plasma samples.

Notwithstanding the above, it should be noted that some clinical validation of the significance of the R263K mutation has already been obtained in the SAILING-clinical trial that compared the use of RAL against DTG in treatment-experienced patients who had undergone previous failures of their therapeutic regimens but who had never before been treated with an integrase inhibitor [15]. The patients in this study all possessed drug resistance mutations that might have compromised the antiviral activity of multiple ARVs in the regimens that they received, but not of the integrase inhibitors and the results showed that DTG was superior to RAL at suppression of viral load in these individuals. In fact, the only drug resistance mutation to have appeared in only two patients in the DTG arm of the study was R263K, whereas failure on the RAL arm of the study led to a broad array of RAL-associated mutations in integrase. Although, the patients who received DTG and who possessed the R263K mutation have apparently continued to be clinically well, new information is needed in regard to mutations that may have developed over time in such individuals, in order to determine whether viral evolution took place to significant extent. Although the data to date suggest that subsequent viral evolution did not take place [13] important questions of durability of responsiveness remain unanswered.

A further thought relates to the reasons for treatment failure in approximately 10%–15% of patients who have received DTG as part of first-line therapy. The most likely reason for this is patient non‑adherence. However, it is inconceivable that all non-adherent individuals who failed DTG failed to take their drugs 100% of the time. Why then did they not develop resistance to DTG as happened in each of the comparator arms in the Single, Flamingo and Spring studies in which patients who failed therapy did develop resistance against each of the nucleoside compounds that were employed in therapy as well as against RAL? In fact, the development of RAL-associated mutations in the Spring study is consistent with the results of other clinical trials in which RAL was used in first-line therapy and in which resistance mutations were identified among RAL failures. Why did the non-adherent patients who received DTG in first-line therapy not generate any resistance mutations to any of the drugs that they received? The only conceivable answer is that they were unable to do so because DTG has the highest barrier to resistance of any anti-HIV compound developed to date. We, of course, believe that the basis for this is the hypothesis outlined in this manuscript. An assessment of clinical specimens from circulating lymphocytes and from lymphocytes present in gut tissue and other body compartments in which HIV is likely to become archived might help to answer this question. It is conceivable that the presence of defective viral forms that contain integrase resistance mutations that relate to the R263K pathway might be much more common than previously thought. However, such defective viruses might not easily be able to grow.

## 4. Dolutegravir and Other Integrase Inhibitors for the Management of HIV-Positive Individuals

Thus, DTG is certainly an agent to consider for patients entering first-line therapy, since the development of R263K and a subsequent mutation may not confer any deleterious effect in regard to patient well-being. In contrast, it is clear that the prior development of mutations associated with resistance against RAL or EVG may compromise the use of DTG in salvage therapy, since each of the Viking I, II, and III studies showed that DTG cannot always be successfully used to salvage patients who were first treated with RAL or EVG and who failed those regimens with resistance-associated mutations [16]. It is also true that some patients who first failed RAL- or EVG-based regimens have responded virologically when treated with DTG as part of second-line therapy, although the durability of the success of DTG in this setting remains to be determined. It is also doubtless true that many patients who have failed RAL and/or EVG may have exhausted many treatment options and that DTG may represent the only reasonable hope for some of these individuals. Nonetheless, it is probably false to believe that integrase inhibitors can or should be used sequentially, beginning with a less potent drug such as RAL or EVG and then switching to DTG; treatment should be initiated with the best drugs that are approved for therapy.

Related to this is that none of the series of secondary mutations to R263K at positions H51Y, M50L, or E138K has ever been shown to restore viral replication capacity, although these may add incrementally to the levels of DTG resistance associated with R263K [17,18,19].

## 5. Conclusions

As stated above, this article makes reference to concepts that should first be studied in animal models such as humanized mice that are infected by HIV or rhesus macaques that are infected by simian immunodeficiency virus (SIV). Although, some clinicians have experimented with monotherapy in the past and are likely to do so again, it is likely that further justification for such studies may first come from clinical trials in which patients are first suppressed with DTG plus two other drugs and then maintained on DTG monotherapy. 

Some might argue that the development of compensatory mutations associated with DTG might only be a matter of time. With each passing day without resistance to DTG in first-line therapy, the hypothesis that has been advanced here becomes more compelling. Among other considerations, it should be noted that failure to develop resistance to DTG or to experience a rebound in viral load in DTG-treated patients could conceivably lead to an inability of people treated with DTG to transmit HIV to others [20,21]. Should this turn out to be the case, there might be profound implications both for future HIV transmission and the sustainability of the HIV epidemic. Such a positive consequence might require that all future HIV-infected persons worldwide be initiated on DTG as a part of first-line therapy.
